# Role of Disulfidptosis in the Local Inflammatory Response of Atopic Dermatitis

**DOI:** 10.1002/clt2.70163

**Published:** 2026-03-23

**Authors:** Dong‐Mei Zhou, Cheng Chen, Yuan‐Fen Liao, Xi‐Meng Ma, Xin‐Hui Gong, Cheng‐Jun Cui, Yu‐Bao Cui

**Affiliations:** ^1^ Clinical Research Center The Affiliated Wuxi People's Hospital of Nanjing Medical University Wuxi Jiangsu Province China; ^2^ Dermatology Department Xishan People's Hospital of Wuxi City Wuxi Branch of Zhongda Hospital Southeast University Wuxi Jiangsu Province China

**Keywords:** atopic dermatitis, disulfidptosis, programmed cell death, skin biopsies, transcriptome analysis

## Abstract

**Background:**

Understanding cell death pathways is critical for elucidating the mechanisms underlying inflammation in atopic dermatitis (AD). This study investigates the role of disulfidptosis, a novel form of programmed cell death, in AD pathogenesis.

**Methods:**

RNA‐seq datasets GSE193309 and GSE121212 from GEO were analyzed to examine 43 disulfidptosis‐related genes. Differentially expressed genes (DEGs) were identified using the limma package. Gene Ontology (GO) and Kyoto Encyclopedia of Genes and Genomes (KEGG) analyses were conducted to annotate biological functions and signaling pathways. Weighted Gene Co‐expression Network Analysis (WGCNA) was applied to identify gene modules significantly associated with AD. A protein‐protein interaction (PPI) network was constructed using STRING data, and further visualized and analyzed with Cytoscape to identify core mRNA‐pathway relationships involving disulfidptosis. Key disulfidptosis‐related mRNAs were validated in the dataset GSE121212. Following house dust mite (HDM) extract treatment, disulfidptosis was induced in HaCaT cells, with characteristic F‐actin collapse visualized by confocal microscopy and redox imbalance confirmed by an increased NADP^+^/NADPH ratio. Expression of disulfidptosis‐related genes was quantified via RT‐qPCR.

**Results:**

A total of 4239, 3570, and 516 DEGs were identified between lesional skin (LS) and healthy control (HC), LS and non‐lesional skin (NL), and NL and HC, respectively. WGCNA clustered these DEGs into 14 co‐expression modules, five of which were significantly correlated with AD. Notably, the turquoise module showed the strongest association with LS and contained four disulfidptosis‐related genes: *ACTB*, *GYS1*, *SLC7A11*, and *MYH9*. These four genes were consistently upregulated in LS compared to both NL and HC. Immunofluorescence staining showed F‐actin contraction and membrane detachment in HDM‐treated HaCaT cells, consistent with disulfidptotic morphology. A significant increase in the NADP^+^/NADPH ratio further supported disulfidptosis induction. qPCR confirmed upregulation of four key disulfidptosis‐related genes (*ACTB*, *GYS1*, *SLC7A11*, and *MYH9*) in response to HDM exposure. Moreover, HDM treatment triggered a pronounced pro‐inflammatory response, as evidenced by the elevated mRNA expression of cytokines IL‐25, IL‐33, TSLP, IL‐6, and IL‐8.

**Conclusion:**

Our findings reveal an association between disulfidptosis‐related gene signatures and AD pathogenesis, suggesting that this pathway may contribute to the inflammatory response observed in lesional skin.

## 1 Introduction

Atopic Dermatitis (AD), frequently referred to as atopic eczema, is a chronic inflammatory disease of the skin marked by itching, rashes, and dry skin. A systematic review discovered that AD affects 17.1% of adults and 22.6% of children annually [1]. The impact of AD is not merely physical but also socio‐economic, with substantial costs being incurred worldwide due to healthcare expenses, morbidity, and quality of life impairments, ultimately contributing significantly to the disease burden of disability.

The heterogeneity of AD is well‐established, with a diverse array of clinical presentations and subtypes. AD is characterized by disrupted epidermal architecture, keratinocyte differentiation anomalies, and T cell‐mediated inflammation [2, 3]. As the body’s primary interface with the environment, the skin is continuously exposed to external stimuli including allergens. The epithelium serves both as a physical barrier and as an active site of cellular responses, including various forms of cell death. A variety of complex factors—including irritants, airborne allergens, food, microorganisms, contact allergens such as HDM, sweat, and scratching—can trigger the onset of symptoms associated with AD [4]. Adult patients with AD often present with more severe skin manifestations and elevated levels of HDM‐specific IgE [4].

Cell death, crucial for normal physiological development and tissue homeostasis, occurs in many forms, most notably apoptosis, necrosis or necroptosis, autophagy, and pyroptosis. Dysregulated apoptosis in AD is remarkable, for example, the recombinant SEB could up‐regulate the expression of tumor necrosis factor alpha (TNFα) in THP‐1 monocyte and induce apoptosis via an extrinsic pathway [5]. In the context of AD, there is a marked expression of GSDMC and GSDMD in keratinocytes derived from lesioned skin, suggesting a potential role for GSDMB‐induced excessive pyroptosis [6]. Similarly, Cathepsin L, which plays a pivotal role in tumor necrosis factors (TNF‐α) induced cell death, shows increased expression in AD patients’ lesional skin [7]. By adjusting the local cytokine and chemokine environment at the site of inflammation, apoptotic and nonapoptotic activation of the Fas/FasL‐dependent signaling pathway may play a significant role in the pathogenesis of AD [8].

Recently, Liu et al. [9] described a novel programmed cell death mechanism, termed “disulfidptosis,” which is distinct from established pathways such as apoptosis, necroptosis, and ferroptosis [10]. Triggered by glucose starvation in cells overexpressing *SLC7A11*, disulfidptosis results from excessive cystine uptake [11]. This causes excessive cystine uptake, leading to abnormal disulfide bonding between F‐actin filaments, cytoskeletal contraction, membrane detachment, and eventual cell death. This leads to aberrant disulfide bonding between F‐actin filaments, causing cytoskeletal contraction, membrane detachment, and cell death. Such disruption of disulfide bond homeostasis not only damages the cytoskeleton but also perturbs the cellular redox balance [9]. Given that F‐actin dynamics maintain cell structure [12] and NADPH serves as a critical antioxidant [13], assessing cytoskeletal integrity and NADPH levels is key to understanding disulfidptosis. In this study, we leveraged skin transcriptome data to investigate the role of disulfidptosis‐related genes, aiming to elucidate how this cell death pathway may contribute to the local inflammatory response in AD pathophysiology.

By uncovering these hitherto unexplored pathways, we hope to improve our understanding of the intricate processes underlying AD and potentially pave the way for innovative therapeutic interventions.

## 2 Materials and Methods

### 2.1 Data Source and Sample Selection

The skin transcriptome datasets GSE193309 and GSE121212 from patients diagnosed with Atopic Dermatitis (AD) were downloaded from the Gene Expression Omnibus (GEO) database [3, 14]. From the GSE193309 dataset, we specifically selected RNA‐seq data extracted from 270 biopsy samples taken from the arm region (Table S1). These samples were divided into a test set (109 samples) and a validation set (161 samples; Table 1). A set of 43 disulfidptosis‐related genes identified in a recent publication by Liu et al. [9] was also extracted for further analysis.

**TABLE 1:** Distribution of arm‐specific samples used in this study.

				HC	LS	NL	Total	
GSE193309	GPL24676	Illumina NovaSeq 6000	Baseline	37	32	40	109	Test set
			3 months	14	17	19	50	Validation set
			6 months	17	11	12	40	
			9 months	15	11	10	36	
			12 months	16	8	11	35	
GSE121212	GPL16791	Illumina HiSeq 2500		38	21	27	86	

*Note:* Samples include HC, LS, and NL from two datasets, GSE193309 and GSE121212, across baseline and follow‐up months.

Abbreviations: HC, Health controls; LS, lesional; NL, nonlesional skin.

### 2.2 Identification of Differentially Expressed mRNAs in Skin Tissue of AD Patients

We employed the “limma” package in R to screen for differentially expressed genes (DEGs) within the 109 test samples from the GSE193309 dataset. This included 37 healthy controls (HC), 32 lesional skin (LS) samples, and 40 non‐lesional skin (NL) samples (Table 1). Significance and false discovery rate (FDR) analyses were conducted, and genes with a *p* < 0.05 and an absolute fold change > 1.5 were deemed statistically significant. This led to the identification of three DEG datasets for AD patients, comparing HC versus LS, HC versus NL, and LS versus NL. Subsequently, we selected disulfidptosis‐related genes following the guidelines established by Liu et al. [9].

### 2.3 Functional Enrichment Analysis

Gene Ontology (GO) analyses were performed on the DEGs to elucidate gene functions, focusing on biological processes, cellular components, and molecular functions. Using Fisher's exact test and multiple comparison tests, we calculated *p* values and false discovery rates (FDR) for each function. We then evaluated the significant function of each DEG. We referred to the Kyoto Encyclopedia of Genes and Genomes (KEGG) database to identify significant signaling pathways. The significance threshold was set at an adjusted *p* value < 0.05 for GO analysis and a *p* value < 0.05 for KEGG analysis.

### 2.4 Weighted Gene Co‐Expression Network Analysis (WGCNA) and Analysis of the AD‐Associated Module

WGCNA was utilized to identify module information from the RNA‐seq data using the “WGCNA” package in R [15]. This involved constructing co‐expression networks from the DEGs. Initially, we generated a correlation matrix for all pairwise correlations and transformed this into a weighted adjacency matrix using a soft‐thresholding power beta = 14. We then created a topological overlap matrix using the blockwiseModules function in R software (parameters: minimum Module Size = 30, module detection sensitivity deepSplit 2, cut height for merging modules 0.2, maxBlockSize = 6000, TOMType = “signed”, corType =  = “pearson”). This allowed us to merge modules with eigengenes correlated above 0.8 to produce co‐expressed modules. A cluster dendrogram was visualized using the plotDendroAndColors function, and the modules were color‐coded based on a dynamic tree cut algorithm [16].

### 2.5 Integrated Bioinformatics Analysis Identifying Disulfidptosis‐Related Genes in Skin Tissue of AD Patients

We selected the disulfidptosis‐related genes and constructed a co‐expression network to understand their regulatory roles. The protein‐protein interaction network (PPI) was constructed using String data. We performed Disulfidptosis‐ mRNA‐ Pathway analysis with CytoScape to understand the involvement of these genes in the pathogenesis of AD.

### 2.6 In Silico‐Validation of Differentially Expressed mRNAs

We selected differentially expressed disulfidptosis‐related genes based on baseline data and submitted these for validation: (1) The comparison among LS, NL, and HC was performed using RNA‐seq data sampled from arms at 3, 6, 9, and 12 months after the baseline visit; (2) These nine disulfidptosis‐related gene expressions were validated using data from the GSE121212 dataset. This rigorous validation step strengthens the conclusions drawn from our analysis.

### 2.7 Immunofluorescence Staining of F‐Actin

Human immortalized keratinocyte HaCaT cells (Zhenjiang Weigen Biotechnology Co. Ltd.) were fixed with 3.7% formaldehyde (prepared in PBS) at room temperature for 20 min. Following fixation, cells were washed three times with PBS containing 0.1% Triton X‐100, with each wash lasting 5 min. F‐actin was stained using Actin‐Tracker Green (Beyotime, C2201s) diluted 1:50 in immunofluorescence secondary antibody diluent (Beyotime, P0108), and incubated at room temperature for 1 h in the dark. After another three washes with PBS containing 0.1% Triton X‐100, the nuclei were counterstained with DAPI (Beyotime, C1002) diluted 1:1000 and incubated for 15 min at room temperature in the dark. The stained cells were then visualized using a confocal microscope (Leica SP8).

### 2.8 Measurement of NADP^+^ and NADPH

HaCaT cells were seeded in 10 cm dishes at a density of 4 × 10^5^ cells per dish. After 24 h of incubation, the cells were divided into two groups: a control group and a treatment group exposed to HDM extract (*Dermatophagoides pteronyssinus*, Greer Laboratories) at a concentration of 20 μg/mL. Following an additional 24 h of treatment, intracellular levels of NADPH and total NADP (NADPH + NADP^+^) were measured using a NADP^+^/NADPH Colorimetric Assay Kit (Elabscience, E‐BC‐K803‐M) according to the manufacturer's instructions.

### 2.9 qPCR Validation of Differentially Expressed mRNAs

HaCaT cells were seeded in six‐well plates at a density of 150,000 cells per well. After 24 h of incubation, the cells were divided into control and treatment groups. The treatment group was exposed to HDM extract (*D. pteronyssinus*, Greer Laboratories) at a final concentration of 20 μg/mL. Following another 24 h of treatment, total RNA was extracted and reverse‐transcribed into cDNA for quantitative real‐time PCR (qPCR) analysis. The primer sequences used for qPCR are listed in Table S2.

### 2.10 Statistical Analysis

Statistical analyses were performed using Prism 9 software (GraphPad Software Inc., La Jolla, USA). An unpaired two‐tailed Student's *t*‐test was used to compare differences between two groups. A *p* value of less than 0.05 was considered statistically significant.

## 3 Results

### 3.1 Differentially Expressed mRNA in Skin Tissue of AD Patients

**FIGURE 1:**
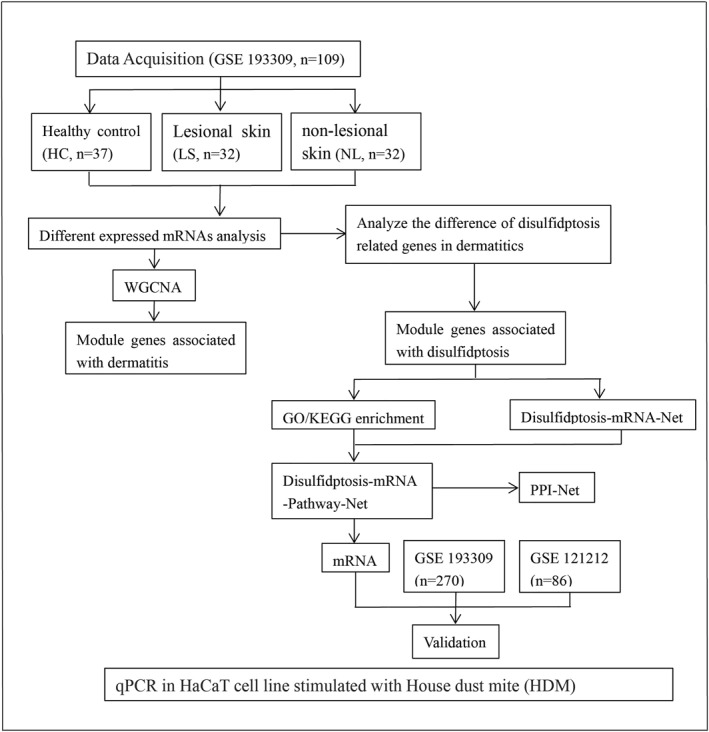
The flow chart shows the overall design of the study.

**FIGURE 2:**
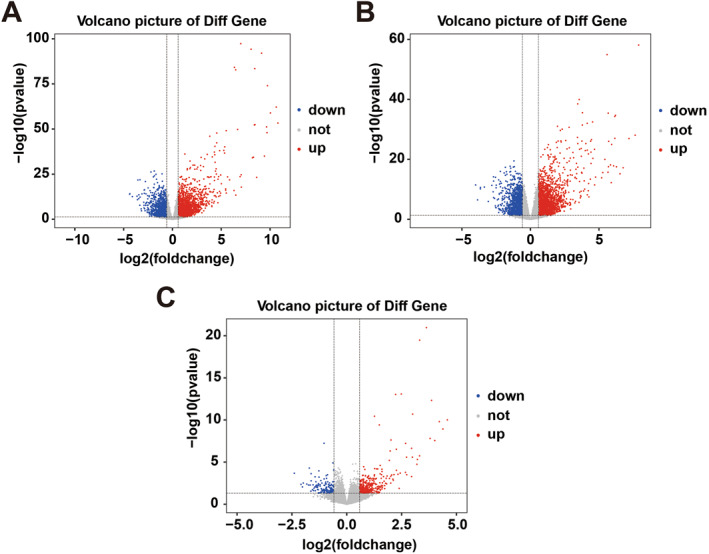
Differential mRNA expression in AD patients’ skin tissue. Volcano plots and represent the significantly differentially expressed genes (DEGs) between LS versus HC (A), NL versus HC (B), LS versus NL (C).

**FIGURE 3:**
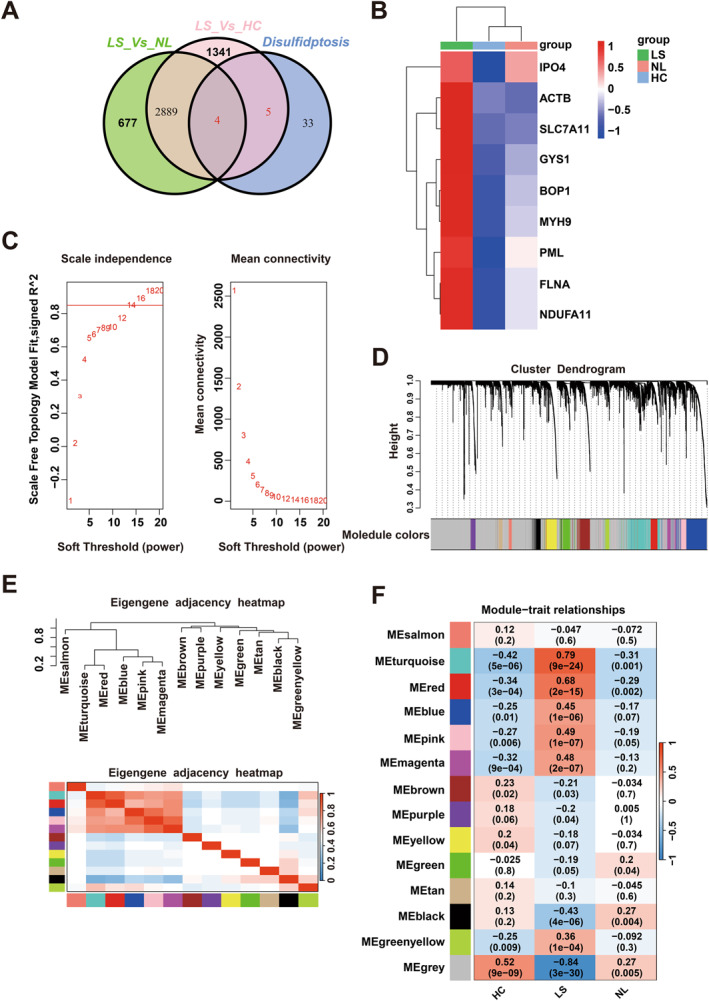
Differentially expressed disulfidptosis‐related genes in AD skin tissue. (A) Venn diagram and (B) heatmaps show nine disulfidptosis‐related genes differentially expressed in LS versus HC, NL versus HC, LS versus NL comparisons. (C) Network topology for different soft‐thresholding powers. The ideal soft‐thresholding power of 14 was determined based on the scale‐free topology criterion. (D) Clustering dendrogram of genes based on consensus topological overlap. Color‐coded modules represent groups of highly connected genes. A total of 14 modules were identified. (E) Adjacency heatmap of module eigengenes. The top panel shows the dendrogram of consensus module eigengenes determined by WGCNA. The red line indicates the merging threshold. The bottom panel visualizes the adjacencies between modules. Red denotes high adjacency (positive correlation) and blue denotes low adjacency (negative correlation). (F) Relationships between modules and samples. Rows denote modules and columns represent samples, with the heatmap displaying the correlation and *p* value. The color gradient from red (1) to blue (−1) signifies the strength of the correlation.

The research design was shown in a flow chart (Figure 1). Utilizing 109 baseline samples from the GSE193309 dataset, we identified 4239, 3570, and 516 mRNAs as differentially expressed between LS and HC, LS and NL, NL and HC, respectively, adhering to a fold change (FC) cutoff value > 1.5 and *p* < 0.05 (Figure 2). A total of 5002 differentially expressed mRNAs were observed among LS, HC and NL. These mRNAs were selected as candidate genes for subsequent WGCNA. Within this differential expression, nine disulfidptosis‐related genes were implicated, specifically ACTB, BOP1, GYS1, SLC7A11, FLNA, IPO4, MYH9, NDUFA11, and PML (Figure 3A,B). Among the differentially expressed disulfidptosis‐related genes, the magnitude of change was most pronounced for SLC7A11 (log_2_FC = 2.34 in LS vs. HC) and MYH9 (log_2_FC = 1.87 in LS vs. HC), while ACTB showed more modest but statistically significant upregulation (log_2_FC = 0.68). These effect sizes are consistent with those reported for established AD‐associated genes such as IL‐13 and CCL17 in the same dataset, supporting the potential biological relevance of these disulfidptosis‐related transcripts.

### 3.2 WGCNA Analysis of mRNAs in Skin Tissue of AD Patients

An mRNA co‐expression network was constructed using the differentially expressed mRNAs in AD patients’ skin tissue. Initially, the top 25% mRNAs were used for cluster analysis, yielding no outlier. Following this, the R package checked the data’s integrity, and a network topology was constructed to determine the soft thresholding power. A soft‐thresholding power beta of 14 was applied to define the adjacency matrix (Figure 3C) under an approximate scale‐free topology criterion. Then, using this power, adjacency and topological matrices were developed. The genes were subsequently clustered via dissimilarity based on the topological matrix, and a dynamic shearing method separated the cluster dendrogram into 14 distinct modules, each distinguished by a different color as per WGCNA convention (Figure 3D).

Of the 5002 selected mRNAs, 2630 did not fit into distinct groups and were allocated to the gray module. The largest module was the turquoise module, followed by the blue module. The module sizes and compositions are depicted in Figure 3C. By generating an eigengene adjacency heatmap (Figure 3E), we confirmed that the regulation directions of these modules were consistent. The 14 modules were classified into two categories, each comprising two branches.

To identify disease‐relevant modules associated with AD phenotypic traits, we evaluated the module‐trait relationships by calculating the biweight midcorrelations between each module eigengene and various disease‐related traits or sample variables (Figure 3F). This heatmap of module‐trait relationships showed correlations between gene expression changes and the modules. Notably, multiple modules, such as turquoise, red, blue, pink, magenta, showed positive correlations with LS (lesional), with correlation coefficients of 0.79, 0.68, 0.45, 0.49, 0.48, respectively.

### 3.3 Functional Enrichment Analysis of AD‐Associated Network Modules

**FIGURE 4:**
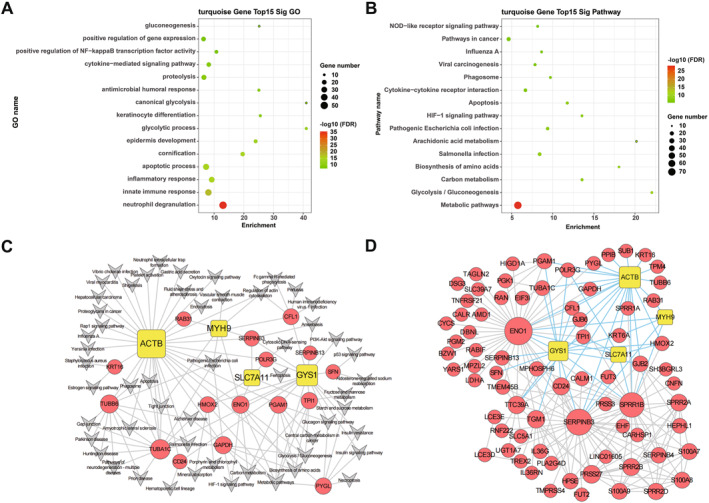
The top 15 significant GO terms (A) and pathways (B) in the turquoise module and protein‐protein interaction (PPI) network for genes. (C) Co‐expression network of disulfidptosis‐related mRNAs in the turquoise module. Circles: mRNAs; rectangles: disulfidptosis‐related genes. Red: upregulated in lesional skin; blue lines: co‐expression relationships. Node size reflects connectivity (larger nodes = greater regulatory capacity); edge thickness indicates correlation strength. (D) Integrated disulfidptosis‐mRNA‐pathway network. Circles: mRNAs; rectangles: disulfidptosis‐related mRNAs; V shapes: enriched pathways; gray polygons: pathway clusters. Node size indicates regulatory capacity.

According to gene counts and module‐sample relationships, genes in the turquoise, blue, black, and red modules were analyzed. The hub genes in these modules are listed in the Tables S1 and S2. Given the inclusion of Disulfidptosis‐mRNA, the GO and pathway analysis of the turquoise module was conducted (Figure 4A,B). The top 15 GO terms included gluconeogenesis, positive regulation of gene expression, positive regulation of NF‐kappaB transcription factor activity, cytokine‐mediated signaling pathway, proteolysis, antimicrobial humoral response, canonical glycolysis, keratinocyte differentiation, glycolytic process, epidermis development, cornification, apoptotic process, inflammatory response, innate immune response, and neutrophil degranulation (Figure 4A). The pathways in the turquoise module encompassed Metabolic pathways, Glycolysis/Gluconeogenesis, Carbon metabolism, Biosynthesis of amino acids, *Salmonella* infection, Arachidonic acid metabolism, Pathogenic *Escherichia coli* infection, HIF‐1 signaling pathway, Apoptosis, Cytokine–cytokine receptor interaction, Phagosome, Viral carcinogenesis, Influenza A, Pathways in cancer, NOD‐like receptor signaling pathway (Figure 4B).

### 3.4 Construction of Disulfidptosis‐mRNA Co‐Expression Network and Disulfidptosis‐mRNA Co‐Expression Pathways

The turquoise module’s co‐expression networks were constructed (Figure 4C), comprising four Disulfidptosis‐mRNAs (ACTB, GYS1, SLC7A11, MYH9). To reveal the possible mechanisms of Disulfidptosis‐mRNA mediated regulation of signaling pathways, significant differential pathways were associated with the turquoise module in the mRNA co‐expression network (Figure 4D), which included four Disulfidptosis mRNAs (ACTB, GYS1, SLC7A11, MYH9) and 13 co‐expression mRNAs (ENO1, TUBA1C, POLR3G, PGAM1, HMOX2, TPI1, RAB31, GAPDH, CD24, SERPINB13, KRT16, CFL1, RAB31).

### 3.5 Disruption of F‐Actin Structure, Altered NADP^+^/NADPH Metabolism, and Pro‐Inflammatory Response in HaCaT Cells Treated With HDM Extract

**FIGURE 5:**
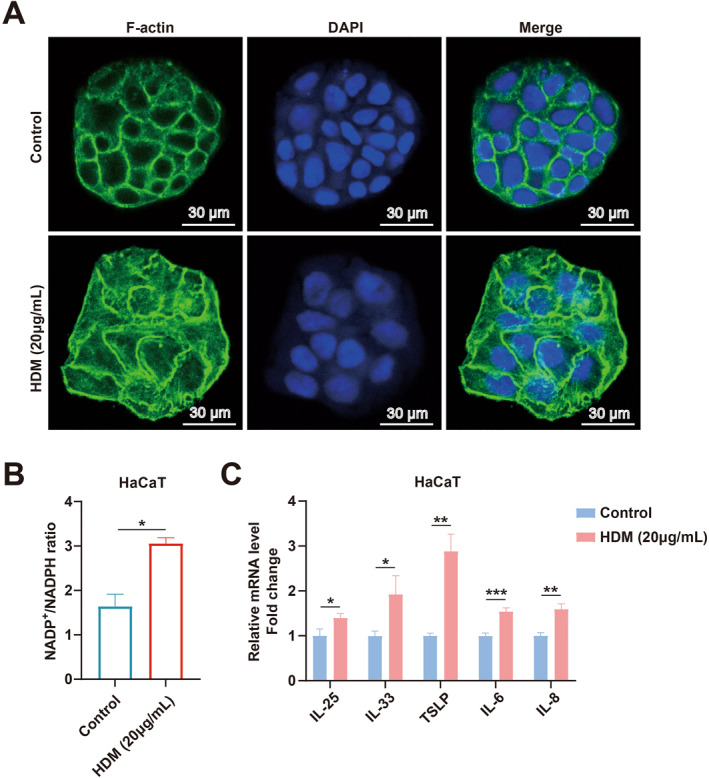
Structural disruption of F‐actin and redox imbalance in HaCaT cells following HDM extract treatment. (A) Immunofluorescence analysis of F‐actin structure after HDM exposure. (B) Metabolic assessment of NADP+/NADPH. *p < 0.05, **p < 0.01, ***p < 0.001. (C) mRNA expression levels of inflammatory cytokines (IL‐25, IL‐33, TSLP, IL‐6, IL‐8) determined by qPCR. Data are presented as mean ± SD; *p < 0.05, **p < 0.01, ***p < 0.001.

Immunofluorescence analysis revealed that, compared with the control group, the F‐actin network in HDM‐treated HaCaT cells was markedly disrupted, exhibiting aggregated and abnormally clustered structures indicative of cytoskeletal disintegration (Figure 5A). Metabolic assessment of the NADP^+^/NADPH ratio showed a significant reduction in NADPH levels after 24 h of HDM exposure, leading to an 86.43% increase in the NADP^+^/NADPH ratio compared to controls (*p* < 0.05), suggesting impaired cellular redox homeostasis (Figure 5B). To further validate the pathophysiological relevance of our model, we characterized the inflammatory cytokine profile upon HDM stimulation. qPCR analysis demonstrated that HDM treatment significantly upregulated the mRNA expression of key AD‐related cytokines, including IL‐25, IL‐33, TSLP, IL‐6, and IL‐8 (Figure 5C). These results collectively demonstrate that HDM stimulation not only induces hallmark features of disulfidptosis but also recapitulates the pro‐inflammatory microenvironment characteristic of AD, thereby strongly supporting the utility and relevance of this in vitro model.

### 3.6 Validation of Differentially Expressed Disulfidptosis‐mRNA

**FIGURE 6:**
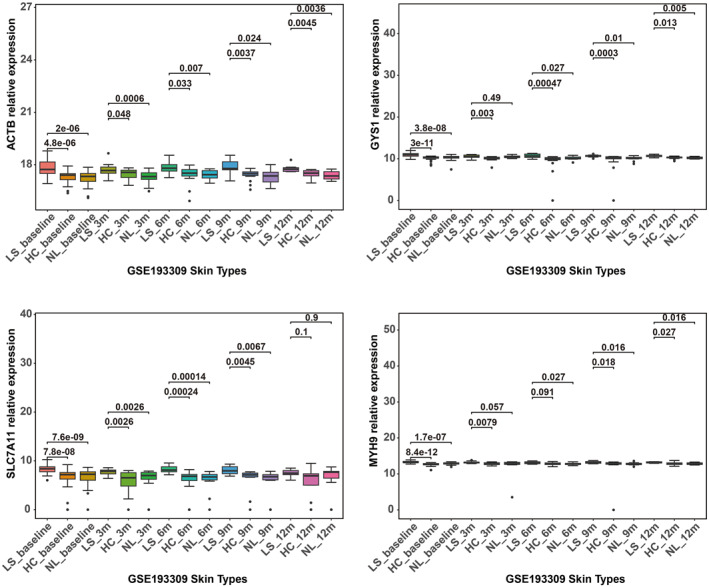
Validation of differentially expressed disulfidptosis‐related mRNAs in the GSE193309 dataset.

**FIGURE 7:**
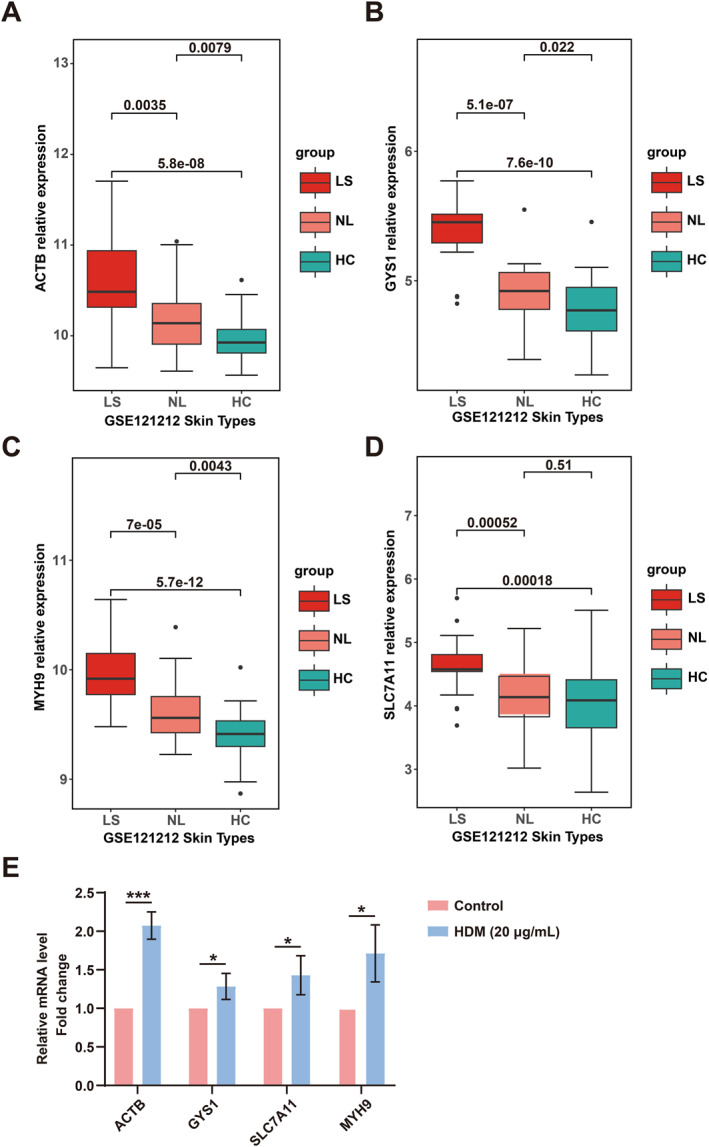
Validation of differentially expressed disulfidptosis‐related mRNAs in the GSE121212 dataset (A–D) and HaCaTs treated with HDM extract (E).

In the GSE193309 dataset, a subpopulation of AD and HC patients underwent a longitudinal study, with skin biopsies taken at 3, 6, 9, and 12 months after the baseline visit. All previous analyses utilized the baseline (109 samples); however, the candidate differentially expressed genes (ACTB, GYS1, SLC7A11, MYH9) were then validated at 3, 6, 9, and 12 months (Figure 6) to compare their expression levels among LS, NL, and HC. Secondly, these four disulfidptosis‐related gene expressions were validated using the GSE121212 dataset (Figure 7A–D). The expression levels of ACTB, GYS1, MYH9, and SLC7A11 were higher in LS than those in NL, HC, reaffirming our initial findings. In a similar manner, the expression levels of these four mRNAs rose in HaCaT cells that were exposed to HDM extract (Figure 7E).

## 4 Discussion

The present study expands our understanding of the molecular signatures underpinning AD. By harnessing small biopsies coupled with omics technologies, a comprehensive transcriptome analysis was performed on a large number of skin samples, sourced from different anatomical regions at various time points. These samples were harvested from lesional, non‐lesional, and healthy skin, enabling a robust investigation of the primary hallmarks of AD, which are recognized as compromised skin barrier integrity and an activated immune response. [17].

Our analysis was primarily focused on the RNA‐seq data extracted from 109 baseline skin biopsy samples from the GSE193309 dataset, which were all collected from the arm region. This investigation led to the identification of 4239, 3570, and 516 differentially expressed mRNAs between lesional and health controls, lesional and non‐lesional skin, non‐lesional skin and health controls, respectively. Notably, there were no significant differences between non‐lesional skin and healthy controls, despite both groups being treated as controls.

Protein‐protein interactions (PPI) are crucial for the majority of biological functions and processes, as most proteins seem to activate their functionalities through these interactions. The PPI network provides a potential role in drug discovery mechanisms. Subsequent gene co‐expression network construction via Weighted Gene Correlation Network Analysis (WGCNA) allowed the categorization of these differentially expressed mRNAs into 14 distinct modules. These modules were identified through the calculation of biweight midcorrelations between each module’s eigengene, a process that unveiled multiple modules associated with AD, including turquoise, red, blue, pink, and magenta. The differentially expressed mRNAs identified in these modules present exciting avenues for future investigation, as they further enrich the molecular signature of AD.

A pivotal advancement in cell death research was the identification of “disulfidptosis” by Xiaoguang Liu et al. [9] a novel programmed cell death mechanism triggered by disulfide stress and characterized by solute carrier family 7 member 11 (*SLC7A11*) overexpression. Under glucose starvation, *SLC7A11* upregulation accelerates cytoplasmic nicotinamide adenine dinucleotide phosphate (NADPH) depletion, leading to aberrant disulfide accumulation, cytoskeletal collapse, and ultimately disulfidptosis [9]. This discovery not only revealed therapeutic opportunities for cancer but also prompted our investigation into its role in inflammatory skin diseases. In this study, we employed house dust mite (HDM) extract—a physiologically relevant in vitro model for atopic dermatitis (AD)—to evaluate disulfidptosis in HaCaT keratinocytes. Unlike TNF‐α/IFN‐γ stimulation, HDM contains proteases (e.g., Der p 1) that directly disrupt epidermal barriers and drive Th2‐polarized inflammation, closely mirroring AD pathophysiology [18, 19]. HDM‐treated HaCaT cells exhibited hallmark features of disulfidptosis, including F‐actin depolymerization, cytoskeletal fragmentation, and a significantly elevated NADP^+^/NADPH ratio, indicative of redox imbalance. Furthermore, HDM stimulation significantly upregulated the expression of key AD‐related cytokines (IL‐25, IL‐33, TSLP, IL‐6, and IL‐8), which are critically involved in the initiation and perpetuation of AD‐associated inflammation. This robust pro‐inflammatory response, coupled with the morphological and biochemical hallmarks of disulfidptosis, provides compelling evidence that our HDM model effectively captures the complex interplay between cell death and inflammation observed in the pathogenesis of AD. These findings collectively suggest that HDM‐induced disulfidptosis may contribute to AD pathogenesis by exacerbating keratinocyte dysfunction within the disease‐specific inflammatory microenvironment.

It is important to distinguish, however, between metabolic reprogramming and definitive disulfidptotic cell death [20]. While our in vitro HDM model demonstrated hallmark features consistent with disulfidptosis—including F‐actin disruption and NADP^+^/NADPH imbalance—the transcriptomic data from patient skin samples indicate altered expression of disulfidptosis‐related genes but cannot confirm active disulfidptosis in vivo. Direct assessment of disulfide stress, quantification of actin cytoskeletal alterations in patient biopsies, and functional validation using specific inhibitors would be required to definitively establish pathway activation in AD tissue.

Inspired by these findings, our study sought to explore the presence and role of disulfidptosis in the context of AD. Among the differentially expressed mRNAs in the GSE193309 dataset, nine disulfidptosis‐related genes were identified (ACTB, BOP1, GYS1, SLC7A11, FLNA, IPO4, MYH9, NDUFA11, and PML). WGCNA analysis clustered these disulfidptosis‐related genes into the turquoise module. The GO analyses of this module included gluconeogenesis, canonical glycolysis, and glycolytic process among the top 15 GO terms.

Given the current understanding of disulfidptosis activation, three conditions must be met: (1) overexpression of SLC7A11, which promotes high uptake of extracellular cysteine and an excessive intracellular accumulation of cysteine, leading to disulfide stress; (2) glucose starvation conditions that block glucose metabolism to generate NADPH via the pentose phosphate pathway (PPP); and (3) formation of aberrant disulfide bonds between actin cytoskeleton proteins [21]. Therefore, gluconeogenesis, canonical glycolysis, and the glycolytic process, all highlighted in our GO analysis, are likely involved in the activation of disulfidptosis. Furthermore, pathway analyses of the turquoise module also included Glycolysis/Gluconeogenesis, indicating the potential activation of disulfidptosis under glucose starvation conditions in AD. Pathway‐based analysis is a powerful tool to identify pathways associated with traits, providing a feasible solution to discover the biological function and mechanism.

It should be noted that our transcriptomic analyses were performed on bulk skin biopsies, which encompass a heterogeneous mixture of cell types including keratinocytes, fibroblasts, and infiltrating immune cells. The observed disulfidptosis‐related gene signatures may therefore reflect contributions from multiple cellular compartments. For instance, while our in vitro experiments focused on keratinocytes, immune cells such as macrophages and T cells—which are known to express SLC7A11 and undergo metabolic reprogramming during activation—could also contribute to the gene expression patterns detected in lesional skin. Future single‐cell RNA sequencing studies would help delineate cell‐type‐specific contributions to disulfidptosis‐related signatures in AD.

Furthering our understanding of the co‐expression networks in the turquoise module, four disulfidptosis‐related mRNAs (ACTB, GYS1, SLC7A11, MYH9) were observed to interact with an additional 13 mRNAs (ENO1, TUBA1C, POLR3G, PGAM1, HMOX2, TPI1, RAB31, GAPDH, CD24, SERPINB13, KRT16, CFL1, RAB31). This intricate interplay of multiple genes further emphasizes the complex molecular landscape that underpins atopic dermatitis (AD).

The first step in the activation of disulfidptosis is the overexpression of SLC7A11, a process previously outlined [9]. Additionally, ACTB, a key component of the actin cytoskeleton, has been highlighted as a hub gene in pediatric AD [22]. This reiterates the crucial role of cytoskeletal alterations in the pathology of AD. Glycogen synthase 1 (GYS1), a pivotal regulator of glycogenesis, is another gene of interest that was overexpressed in this module. Overexpression of GYS1 suggests that glycolytic and glycogen synthesis mechanisms are upregulated, an observation in line with previous findings [23]. Also overexpressed was the MYH9 gene, which encodes non‐muscle myosin heavy chain IIA (NMMHC‐IIA). This gene has been linked with a group of autosomal, dominantly inherited disorders, underscoring the impact of aberrant MYH9 expression on various disease states [24].

Notably, all four of these disulfidptosis‐related genes were significantly overexpressed in lesional skin compared to non‐lesional skin and healthy controls. This overexpression was observed in samples taken 3, 6, 9, and 12 months after the baseline visit, suggesting a persistently altered expression pattern in AD. This increased expression was additionally supported by another set of data, GSE121212, and by keratinocytes treated with HDM, which are linked to skin inflammation in AD [19, 25]. This further solidified the evidence for the involvement of these genes in the development of AD.

Interestingly, metabolic reprogramming and cytoskeletal alterations have been implicated in other inflammatory dermatoses. For example, psoriasis lesions exhibit upregulated glycolysis‐related genes (including GAPDH and ENO1) and actin cytoskeleton remodeling, while cutaneous T‐cell lymphoma shows altered SLC7A11 expression and redox balance [26, 27]. The disulfidptosis‐related signatures identified in our study—particularly the upregulation of ACTB, GYS1, SLC7A11, and MYH9—may therefore represent a broader inflammatory response pattern rather than an AD‐specific mechanism. Comparative transcriptomic analyses across inflammatory skin conditions would help determine whether these signatures are uniquely enriched in AD or reflect common pathways activated in chronic skin inflammation.

The other mRNAs identified in the turquoise module (ENO1, TUBA1C, POLR3G, PGAM1, HMOX2, TPI1, RAB31, GAPDH, CD24, SERPINB13, KRT16, CFL1, RAB31), which interact with the disulfidptosis‐related mRNAs, could provide significant insights into the underlying mechanisms of AD. These interacting genes represent potential targets for further research, and their interactions may shed light on the intricate network of molecular events that contribute to AD.

Although our qPCR validation confirmed upregulation of the four disulfidptosis‐related genes at the mRNA level in HDM‐treated keratinocytes, protein‐level confirmation in human AD tissue remains to be established. Immunohistochemical analysis of ACTB, GYS1, SLC7A11, and MYH9 protein expression and localization in patient biopsies would provide important validation of the translational relevance of our findings. Furthermore, functional studies using genetic or pharmacological manipulation of these genes in relevant cell types would be necessary to establish causal relationships.

While our findings elucidate the potential involvement of disulfidptosis in AD pathogenesis through transcriptomic and in vitro analyses, certain limitations should be acknowledged. Our study provides mechanistic insights using transcriptomic data and in vitro models, but we acknowledge that validation in clinical samples or animal models would strengthen the translational relevance. Future studies will utilize AD mouse models to confirm the role of disulfidptosis in vivo, particularly in keratinocyte‐immune cell crosstalk.

## 5 Conclusion

This study identifies disulfidptosis‐related gene signatures upregulated in AD lesional skin and linked to metabolic and cytoskeletal pathways. In HDM‐stimulated keratinocytes, we observed features consistent with disulfidptosis alongside a pro‐inflammatory cytokine response. These findings suggest a potential role for disulfidptosis‐related pathways in the AD inflammatory microenvironment, though definitive evidence in patient tissue remains lacking. Future studies should include protein‐level validation, cell‐type‐specific analysis using single‐cell approaches, and functional interrogation of candidate genes in vivo to assess whether targeting disulfidptosis may offer a therapeutic strategy for AD.

## Author Contributions

**Dong‐Mei Zhou:** investigation, methodology, formal analysis, writing – original draft, software. **Cheng Chen:** investigation, methodology, formal analysis, funding acquisition, software. **Yuan‐Fen Liao:** investigation, data curation, validation, visualization. **Xi‐Meng Ma:** investigation, methodology, resources. **Xin‐Hui Gong:** investigation, methodology, resources. **Cheng‐Jun Cui:** writing – original draft, project administration, supervision, validation. **Yu‐Bao Cui:** conceptualization, supervision, project administration, funding acquisition, writing – review and editing

## Funding

This study was supported by the “333 project” of Jiangsu Province in 2022 (ZUZHIBU 202221001), the Top Talents Project of the Wuxi Taihu Lake Talent Plan (2020THRC‐GD‐7), and the Project of Wuxi Health Commission (Grant q201716).

## Ethics Statement

The authors have nothing to report.

## Consent

All the authors have read and agreed to the published version of the manuscript.

## Conflicts of Interest

The authors declare no conflicts of interest.

## Supporting information


**Table S1:** Clinical data of the patients from GSE193309 dataset.

**Table S2:** The primers sequences used in qPCR.

## Data Availability

The data that support the findings of this study are openly available in GEO at https://www.ncbi.nlm.nih.gov/geo/query/acc.cgi?acc=GSE193309 (reference number GSE193309) and https://www.ncbi.nlm.nih.gov/geo/query/acc.cgi?acc=GSE121212 (reference number GSE121212).
