# Supplementary material for: Role of Disulfidptosis in the Local Inflammatory Response of Atopic Dermatitis
Source: Clin Transl Allergy. 2026 Mar 23;16(3):e70163. doi: 10.1002/clt2.70163 (PMC13093619; doi:10.1002/clt2.70163)
Supplement: Supplementary file 1 — Table S1: Clinical data of the patients from GSE193309 dataset. [file CLT2-16-e70163-s001.docx]

**Supplementary Table 1.** Clinical data of the patients from GSE193309 dataset

| subjects | skin type | gender | scorad | easi | anatomic_region |
| --- | --- | --- | --- | --- | --- |
| 1 | NL | female | 17.6 | 1.5 | arm |
| 2 | LS | female | 48 | 5.2 | arm |
| 3 | NL | female | 48 | 5.2 | arm |
| 4 | LS | female | 48 | 5.2 | arm |
| 5 | NL | female | 48 | 5.2 | arm |
| 6 | LS | female | 15.5 | 1.2 | arm |
| 7 | LS | female | 15.5 | 1.2 | arm |
| 8 | NL | female | 15.5 | 1.2 | arm |
| 9 | NL | female | 15.5 | 1.2 | arm |
| 10 | LS | female | 23.4 | 2.4 | arm |
| 11 | NL | female | 23.4 | 2.4 | arm |
| 12 | LS | female | 23.4 | 2.4 | arm |
| 13 | LS | female | 25.8 | 1.9 | arm |
| 14 | NL | female | 25.8 | 1.9 | arm |
| 15 | LS | female | 25.8 | 1.9 | arm |
| 16 | NL | female | 23.9 | 2.5 | arm |
| 17 | LS | female | 23.9 | 2.5 | arm |
| 18 | LS | female | 23.9 | 2.5 | arm |
| 19 | NL | female | 57 | 21.6 | arm |
| 20 | NL | female | 57 | 21.6 | arm |
| 21 | LS | female | 57 | 21.6 | arm |
| 22 | LS | female | 57 | 21.6 | arm |
| 23 | LS | female | 67.6 | 22.3 | arm |
| 24 | NL | female | 67.6 | 22.3 | arm |
| 25 | LS | female | 67.6 | 22.3 | arm |
| 26 | NL | female | 28.9 | 1.4 | arm |
| 27 | NL | female | 28.9 | 1.4 | arm |
| 28 | NL | female | 40.6 | 6.4 | arm |
| 29 | LS | female | 40.6 | 6.4 | arm |
| 30 | NL | female | 40.6 | 6.4 | arm |
| 31 | LS | female | 40.6 | 6.4 | arm |
| 32 | LS | female | 29.1 | 2.4 | arm |
| 33 | NL | female | 29.1 | 2.4 | arm |
| 34 | LS | female | 29.1 | 2.4 | arm |
| 35 | NL | female | 29.1 | 2.4 | arm |
| 36 | LS | female | 17.3 | 1.2 | arm |
| 37 | NL | female | 17.3 | 1.2 | arm |
| 38 | LS | female | 17.3 | 1.2 | arm |
| 39 | NL | female | 17.3 | 1.2 | arm |
| 40 | LS | female | 39.3 | 1.6 | arm |
| 41 | NL | female | 39.3 | 1.6 | arm |
| 42 | NL | female | 39.3 | 1.6 | arm |
| 43 | LS | female | 39.3 | 1.6 | arm |
| 44 | LS | female | 26.8 | 0.8 | arm |
| 45 | NL | female | 26.8 | 0.8 | arm |
| 46 | NL | female | 26.8 | 0.8 | arm |
| 47 | LS | female | 14.1 | 0.8 | arm |
| 48 | NL | female | 14.1 | 0.8 | arm |
| 49 | LS | female | 14.1 | 0.8 | arm |
| 50 | LS | female | 32.7 | 9.7 | arm |
| 51 | NL | female | 32.7 | 9.7 | arm |
| 52 | LS | female | 32.7 | 9.7 | arm |
| 53 | NL | female | 32.7 | 9.7 | arm |
| 54 | NL | female | 19.6 | 0.9 | arm |
| 55 | LS | female | 19.6 | 0.9 | arm |
| 56 | LS | female | 19.6 | 0.9 | arm |
| 57 | NL | female | 19.6 | 0.9 | arm |
| 58 | LS | female | 7.4 | 0 | arm |
| 59 | NL | female | 7.4 | 0 | arm |
| 60 | LS | female | 7.4 | 0 | arm |
| 61 | NL | female | 7.4 | 0 | arm |
| 62 | LS | female | 61 | 8.6 | arm |
| 63 | LS | female | 61 | 8.6 | arm |
| 64 | NL | female | 61 | 8.6 | arm |
| 65 | NL | female | 61 | 8.6 | arm |
| 66 | LS | female | 78.9 | 37.2 | arm |
| 67 | NL | female | 11.3 | 1 | arm |
| 68 | NL | female | 11.3 | 1 | arm |
| 69 | NL | female | 12.8 | 0.6 | arm |
| 70 | LS | female | 55.7 | 9.6 | arm |
| 71 | NL | female | 55.7 | 9.6 | arm |
| 72 | NL | female | 55.7 | 9.6 | arm |
| 73 | LS | female | 55.7 | 9.6 | arm |
| 74 | NL | female | 24.6 | 2.6 | arm |
| 75 | LS | female | 24.6 | 2.6 | arm |
| 76 | LS | female | 24.6 | 2.6 | arm |
| 77 | NL | female | 24.6 | 2.6 | arm |
| 78 | NL | female | 44.6 | 1.2 | arm |
| 79 | LS | female | 44.6 | 1.2 | arm |
| 80 | LS | female | 29.8 | 2.3 | arm |
| 81 | LS | female | 29.8 | 2.3 | arm |
| 82 | NL | female | 29.8 | 2.3 | arm |
| 83 | NL | female | 35.6 | 6.7 | arm |
| 84 | NL | female | 35.6 | 6.7 | arm |
| 85 | LS | female | 44.6 | 1.2 | arm |
| 86 | NL | female | 45.2 | 1.5 | arm |
| 87 | LS | female | 45.2 | 1.5 | arm |
| 88 | LS | female | 45.2 | 1.5 | arm |
| 89 | NL | female | 45.2 | 1.5 | arm |
| 90 | NL | female | 53.7 | 3 | arm |
| 91 | NL | female | 53.7 | 3 | arm |
| 92 | LS | female | 26.9 | 0.8 | arm |
| 93 | NL | female | 26.9 | 0.8 | arm |
| 94 | NL | female | 26.9 | 0.8 | arm |
| 95 | LS | female | 34.1 | 1.7 | arm |
| 96 | NL | female | 34.1 | 1.7 | arm |
| 97 | NL | female | 34.1 | 1.7 | arm |
| 98 | NL | female | 46.3 | 1.7 | arm |
| 99 | LS | female | 46.3 | 1.7 | arm |
| 100 | NL | female | 46.3 | 1.7 | arm |
| 101 | LS | male | 12.5 | 1 | arm |
| 102 | LS | male | 12.5 | 1 | arm |
| 103 | NL | male | 12.5 | 1 | arm |
| 104 | NL | male | 12.5 | 1 | arm |
| 105 | LS | male | 12.5 | 0.5 | arm |
| 106 | NL | male | 12.5 | 0.5 | arm |
| 107 | NL | male | 12.5 | 0.5 | arm |
| 108 | LS | male | 12.5 | 0.5 | arm |
| 109 | LS | male | 9 | 0.5 | arm |
| 110 | NL | male | 9 | 0.5 | arm |
| 111 | LS | male | 9 | 0.5 | arm |
| 112 | NL | male | 9 | 0.5 | arm |
| 113 | LS | male | 19.5 | 1.7 | arm |
| 114 | NL | male | 19.5 | 1.7 | arm |
| 115 | NL | male | 19.5 | 1.7 | arm |
| 116 | LS | male | 19.5 | 1.7 | arm |
| 117 | LS | male | 10.7 | 0.4 | arm |
| 118 | NL | male | 10.7 | 0.4 | arm |
| 119 | LS | male | 10.7 | 0.4 | arm |
| 120 | NL | male | 10.7 | 0.4 | arm |
| 121 | LS | female | 30.4 | 4 | arm |
| 122 | NL | female | 30.4 | 4 | arm |
| 123 | LS | female | 30.4 | 4 | arm |
| 124 | NL | female | 42.4 | 1.2 | arm |
| 125 | NL | female | 42.4 | 1.2 | arm |
| 126 | LS | male | 32.7 | 1.6 | arm |
| 127 | NL | male | 32.7 | 1.6 | arm |
| 128 | NL | male | 32.7 | 1.6 | arm |
| 129 | NL | female | 12.8 | 0.2 | arm |
| 130 | NL | female | 12.8 | 0.2 | arm |
| 131 | LS | female | 12.8 | 0.2 | arm |
| 132 | NL | female | 23.5 | 0.7 | arm |
| 133 | LS | female | 23.5 | 0.7 | arm |
| 134 | NL | female | 23.5 | 0.7 | arm |
| 135 | LS | female | 23.5 | 0.7 | arm |
| 136 | NL | female | 12.9 | 0.4 | arm |
| 137 | LS | female | 12.9 | 0.4 | arm |
| 138 | LS | female | 19.2 | 0.5 | arm |
| 139 | NL | female | 19.2 | 0.5 | arm |
| 140 | NL | female | 19.2 | 0.5 | arm |
| 141 | NL | male | 45 | 4.2 | arm |
| 142 | LS | male | 45 | 4.2 | arm |
| 143 | NL | male | 45 | 4.2 | arm |
| 144 | LS | male | 45 | 4.2 | arm |
| 145 | LS | male | 47.2 | 4.5 | arm |
| 146 | LS | male | 47.2 | 4.5 | arm |
| 147 | NL | male | 47.2 | 4.5 | arm |
| 148 | NL | male | 47.2 | 4.5 | arm |
| 149 | NL | male | 20.6 | 3 | arm |
| 150 | LS | male | 20.6 | 3 | arm |
| 151 | LS | male | 20.6 | 3 | arm |
| 152 | NL | male | 20.6 | 3 | arm |
| 153 | LS | male | 20.6 | 3 | arm |
| 154 | NL | male | 20.6 | 3 | arm |
| 155 | NL | male | 20.6 | 3 | arm |
| 156 | LS | male | 20.6 | 3 | arm |
| 157 | NL | male | 10.8 | 0.7 | arm |
| 158 | LS | male | 10.8 | 0.7 | arm |
| 159 | LS | male | 10.8 | 0.7 | arm |
| 160 | NL | female | NA | 0.6 | arm |
| 161 | LS | female | NA | 0.6 | arm |
| 162 | NL | female | NA | 0.6 | arm |
| 163 | NL | female | 35.4 | 1.6 | arm |
| 164 | NL | female | 35.4 | 1.6 | arm |
| 165 | LS | male | 49.8 | 6.4 | arm |
| 166 | NL | male | 49.8 | 6.4 | arm |
| 167 | NL | male | 49.8 | 6.4 | arm |
| 168 | LS | female | 27 | 1.9 | arm |
| 169 | NL | female | 27 | 1.9 | arm |
| 170 | LS | female | 27 | 1.9 | arm |
| 171 | NL | female | 27 | 1.9 | arm |
| 172 | HC | female | NA | NA | arm |
| 173 | HC | female | NA | NA | arm |
| 174 | HC | female | NA | NA | arm |
| 175 | HC | female | NA | NA | arm |
| 176 | HC | female | NA | NA | arm |
| 177 | HC | female | NA | NA | arm |
| 178 | HC | female | NA | NA | arm |
| 179 | HC | female | NA | NA | arm |
| 180 | HC | female | NA | NA | arm |
| 181 | HC | female | NA | NA | arm |
| 182 | HC | female | NA | NA | arm |
| 183 | HC | female | NA | NA | arm |
| 184 | HC | female | NA | NA | arm |
| 185 | HC | female | NA | NA | arm |
| 186 | HC | female | NA | NA | arm |
| 187 | HC | male | NA | NA | arm |
| 188 | HC | male | NA | NA | arm |
| 189 | HC | female | NA | NA | arm |
| 190 | HC | female | NA | NA | arm |
| 191 | HC | female | NA | NA | arm |
| 192 | HC | female | NA | NA | arm |
| 193 | HC | female | NA | NA | arm |
| 194 | HC | female | NA | NA | arm |
| 195 | HC | female | NA | NA | arm |
| 196 | HC | female | NA | NA | arm |
| 197 | HC | female | NA | NA | arm |
| 198 | HC | female | NA | NA | arm |
| 199 | HC | female | NA | NA | arm |
| 200 | HC | female | NA | NA | arm |
| 201 | HC | female | NA | NA | arm |
| 202 | HC | female | NA | NA | arm |
| 203 | HC | female | NA | NA | arm |
| 204 | HC | female | NA | NA | arm |
| 205 | HC | female | NA | NA | arm |
| 206 | HC | female | NA | NA | arm |
| 207 | HC | female | NA | NA | arm |
| 208 | HC | female | NA | NA | arm |
| 209 | HC | female | NA | NA | arm |
| 210 | HC | female | NA | NA | arm |
| 211 | HC | female | NA | NA | arm |
| 212 | HC | female | NA | NA | arm |
| 213 | HC | female | NA | NA | arm |
| 214 | HC | female | NA | NA | arm |
| 215 | HC | female | NA | NA | arm |
| 216 | HC | female | NA | NA | arm |
| 217 | HC | female | NA | NA | arm |
| 218 | HC | female | NA | NA | arm |
| 219 | HC | female | NA | NA | arm |
| 220 | HC | female | NA | NA | arm |
| 221 | HC | female | NA | NA | arm |
| 222 | HC | female | NA | NA | arm |
| 223 | HC | female | NA | NA | arm |
| 224 | HC | female | NA | NA | arm |
| 225 | HC | female | NA | NA | arm |
| 226 | HC | female | NA | NA | arm |
| 227 | HC | female | NA | NA | arm |
| 228 | HC | female | NA | NA | arm |
| 229 | HC | female | NA | NA | arm |
| 230 | HC | female | NA | NA | arm |
| 231 | HC | female | NA | NA | arm |
| 232 | HC | female | NA | NA | arm |
| 233 | HC | female | NA | NA | arm |
| 234 | HC | female | NA | NA | arm |
| 235 | HC | female | NA | NA | arm |
| 236 | HC | female | NA | NA | arm |
| 237 | HC | female | NA | NA | arm |
| 238 | HC | female | NA | NA | arm |
| 239 | HC | female | NA | NA | arm |
| 240 | HC | male | NA | NA | arm |
| 241 | HC | male | NA | NA | arm |
| 242 | HC | female | NA | NA | arm |
| 243 | HC | female | NA | NA | arm |
| 244 | HC | female | NA | NA | arm |
| 245 | HC | female | NA | NA | arm |
| 246 | HC | female | NA | NA | arm |
| 247 | HC | female | NA | NA | arm |
| 248 | HC | female | NA | NA | arm |
| 249 | HC | female | NA | NA | arm |
| 250 | HC | female | NA | NA | arm |
| 251 | HC | female | NA | NA | arm |
| 252 | HC | female | NA | NA | arm |
| 253 | HC | female | NA | NA | arm |
| 254 | HC | female | NA | NA | arm |
| 255 | HC | female | NA | NA | arm |
| 256 | HC | female | NA | NA | arm |
| 257 | HC | female | NA | NA | arm |
| 258 | HC | female | NA | NA | arm |
| 259 | HC | female | NA | NA | arm |
| 260 | HC | male | NA | NA | arm |
| 261 | HC | male | NA | NA | arm |
| 262 | HC | male | NA | NA | arm |
| 263 | HC | male | NA | NA | arm |
| 264 | HC | male | NA | NA | arm |
| 265 | HC | male | NA | NA | arm |
| 266 | HC | male | NA | NA | arm |
| 267 | HC | male | NA | NA | arm |
| 268 | HC | male | NA | NA | arm |
| 269 | HC | male | NA | NA | arm |
| 270 | HC | male | NA | NA | arm |
