# Supplementary material for: Role of Disulfidptosis in the Local Inflammatory Response of Atopic Dermatitis
Source: Clin Transl Allergy. 2026 Mar 23;16(3):e70163. doi: 10.1002/clt2.70163 (PMC13093619; doi:10.1002/clt2.70163)
Supplement: Supplementary file 2 — Table S2: The primers sequences used in qPCR. [file CLT2-16-e70163-s002.docx]

**Supplementary Table 2** The primers sequences used in qPCR.

| Gene name | Forward (5′-3′) | Reverse (5′-3′) |
| --- | --- | --- |
| ACTB | GGCCAACCGCGAGAAGATGAC | GGATAGCACAGCCTGGATAGCAAC |
| GYS1 | GCCGCTATGAGTTCTCCAACAAGG | GTCTGCTCGCTGCCGTTCAC |
| SLC7A11 | ACGGTGGTGTGTTTGCTGTCTC | GCTGGTAGAGGAGTGTGCTTGC |
| MYH9 | GAATGACAACTCCTCCCGCTTCG | GGTCCGTTCTTCCTTGGCTTGG |
| IL-25 | CAAGTGGAGTGAGAAACTGGGATC | AGTGGCTGTAGGTGTGGGTTC |
| IL-33 | GGTGACGGTGTTGATGGTAAGATG | AGAGTGTTCCTTGTTGTTGGCATG |
| TSLP | AAATCCAGAGCCTAACCTTCAATCC | CCTCTTCTTCATTGCCTGAGTAGC |
| IL-6 | CAAAGAGGCACTGGCAGAAA | CTGCACAGCTCTGGCTTGTT |
| IL-8 | CTTGGCAGCCTTCCTGATTT | TTCCTTGGGGTCCAGACAGA |
| GAPDH | GAGTCAACGGATTTGGTCGT | GACAAGCTTCCCGTTCTCAG |
